# Inverse association between the extracellular matrix protein Tenascin-X and concurrent abdominal aortic calcification in patients with primary aldosteronism

**DOI:** 10.3389/fendo.2026.1900237

**Published:** 2026-10-01

**Authors:** Rangrang Zhang, Mubareke Aila, Qing Zhu, Saibire Saiding, Menghui Wang, Ting Wu, Bingxuan Guo, Nanfang Li, Qin Luo

**Affiliations:** Hypertension Center of People’s Hospital of Xinjiang Uygur Autonomous Region, Xinjiang Hypertension Institute, National Health Committee Key Laboratory of Hypertension Clinical Research Key Laboratory of Xinjiang Uygur Autonomous Region, Hypertension Research Laboratory, Xinjiang Clinical Medical Research Center for Hypertension (Cardio-Cerebrovascular) Diseases, Urumqi, China

**Keywords:** abdominal aortic calcification, hypertension, primary aldosteronism, proteomics, tenascin-X

## Abstract

**Background:**

Patients diagnosed with primary aldosteronism (PA) are at significantly increased risk of abdominal aortic calcification (AAC) and subsequent cardiovascular complications. Notably, abdominal aortic calcification is independently associated with adverse cardiovascular outcomes. However, to date, no serum proteomic profiling has been specially carried out in PA patients complicated with AAC, leaving an unaddressed research gap. This study was designed to identify serum differentially expressed proteins (DEPs) via DIA-based untargeted proteomics.

**Methods:**

In this translational research project, data-independent acquisition (DIA) mass spectrometry was utilized to contrast serum proteomic landscapes from two PA subgroups: patients presenting with AAC and those without the calcification lesion. GO and KEGG functional enrichment analyses were conducted to decode biological functions of screened DEPs. Tenascin-X (TNX), an extracellular matrix molecule encoded by the *TNXB* gene, was selected as the core candidate and validated via aldosterone-exposed vascular smooth muscle cells (VSMCs) as well as aldosterone-intervened mice.

**Results:**

In total, 38 DEPs were distinguished across the two patient cohorts, comprising 6 upregulated proteins and 32 proteins with lowered expression levels. Gene Ontology results illustrated these molecules predominantly reside within extracellular regions and take part in cellular adhesion and immune regulatory activities. KEGG pathway enrichment further highlighted prominent enrichment within the extracellular matrix receptor interaction cascade. Markedly suppressed TNX expression was detected in aldosterone-treated VSMCs (*P* < 0.001). In mouse aortic specimens, merely a marginal declining tendency of TNX was observed, which failed to reach statistical significance (*P* = 0.054).

**Conclusion:**

This proteomic study analyzes serum protein profiles in primary aldosteronism patients with and without abdominal aortic calcification, and identifies reduced serum TNX expression in PA patients complicated by abdominal aortic calcification. Further cellular experiments and aldosterone-intervened mouse studies revealed downregulation or a downward trend of key candidate molecule TNX, providing partial preliminary experimental support for the proteomic-identified candidate TNX. This study offers new research ideas for subsequent mechanistic investigations into abdominal aortic calcification in patients with primary aldosteronism.

## 1 Introduction

Hypertension is a prevalent chronic condition that significantly impacts human health, affecting 31.1% of adults worldwide (1). In China, the number of hypertension cases is projected to reach 1.56 billion by 2025 (2, 3). Numerous studies have demonstrated that secondary hypertension poses a greater risk due to its more severe onset and more extensive target organ damage compared to essential hypertension (EH). Early screening and diagnosis are essential for effective management.

Primary aldosteronism (PA) is an endocrine cause of hypertension characterized by a clinical syndrome of hypertension, with or without hypokalemia, hyperaldosteronism, and hyponephrinemia. The prevalence of PA among patients with grade 1, 2, and 3 hypertension is 1.99%, 8.02%, and 13.2%, respectively (4), while the prevalence of PA in individuals with refractory hypertension ranges from 17% to 23% (5).

Previous studies have identified abdominal aortic calcification (AAC) as a significant risk factor for cardiovascular mortality (6). A meta-analysis demonstrated that patients with vascular calcification face a 3–4 times higher risk of mortality and cardiovascular events compared to those without (7). The increased risk of cardiovascular events (8) and mortality in patients with PA compared to those with essential hypertension (EH) (9) suggests that cardiovascular damage in PA patients occurs earlier and is more severe, likely due to factors such as arterial stiffness, calcification, and widespread tissue inflammation and fibrosis (10). Liu et al. (11) found that the incidence of AAC in PA patients was approximately twice as high as in age-, sex-, and blood pressure-matched EH patients. Pathological studies have categorized PA-related vascular calcification as either intimal or medial, depending on the site of involvement (12). Accumulating evidence indicates that vascular calcification (VC) constitutes an active, cell-driven process characterized by the osteogenic differentiation of vascular smooth muscle cells (VSMCs) (13). Given the limited understanding of the specific mechanisms of AAC in PA, further research is crucial to elucidate these pathways and facilitate early intervention.

Mounting evidence confirms aldosterone triggers vascular extracellular matrix (ECM) remodeling through mineralocorticoid receptors, exerting pro-oxidant, pro-inflammatory and profibrotic effects that disrupt collagen turnover balance (14) (15). Proteomic data from aldosterone-producing adenomas further proves hyperaldosteronism disturbs ECM signaling networks (16). Disordered ECM accelerates vascular smooth muscle osteogenic transformation and aortic mineral deposition (17–19). Despite separate reports on the two pathways, no serum ECM protein profiles are available for PA patients with AAC. Conventional DDA proteomics poorly detects low-abundance circulating ECM proteins, whereas Data-independent acquisition (DIA) untargeted proteomics provides superior coverage and quantification to screen serum differentially expressed proteins related to early AAC in PA populations.

DIA, a label-free proteomic technique, offers superior coverage, accuracy, and reproducibility compared to conventional methods. In this study, DIA was employed to screen serum differentially expressed proteins in patients with primary PA and AAC. To our knowledge, no proteomic studies have specifically addressed AAC in PA patients. Therefore, we employed DIA technology to compare serum differentially expressed proteins (DEPs) between PA patients with and without AAC, integrated with bioinformatics analysis to elucidate the key signaling pathways involved in AAC development in PA. Subsequently, critical DEPs were selected for further validation through *in vivo* and *in vitro* experiments.

## 2 Methods

### 2.1 Study population

This single-center retrospective case-control study was conducted at the Hypertension Diagnosis and Treatment Center between November 2020 and May 2022. Thirty patients with a confirmed diagnosis of PA were initially enrolled and divided into two groups: PA with abdominal aortic calcification (AAC, n=15) and PA without AAC (n=15). The two groups were pre-planned for 1:1 matching according to age and duration of hypertension.

Exclusion criteria included current or history of smoking, primary hyperparathyroidism, diabetes mellitus, autoimmune diseases, systemic vasculitis, malignant tumors, severe hepatic, renal, cardiac or pulmonary insufficiency, recent trauma, infection, surgery or stress conditions, and pregnancy or lactation. After one hemolyzed sample was excluded from the PA without AAC group, final sample sizes were 15 (PA-AAC) and 14 (PA-non-AAC)(**Figure 1**).

**Figure 1:**
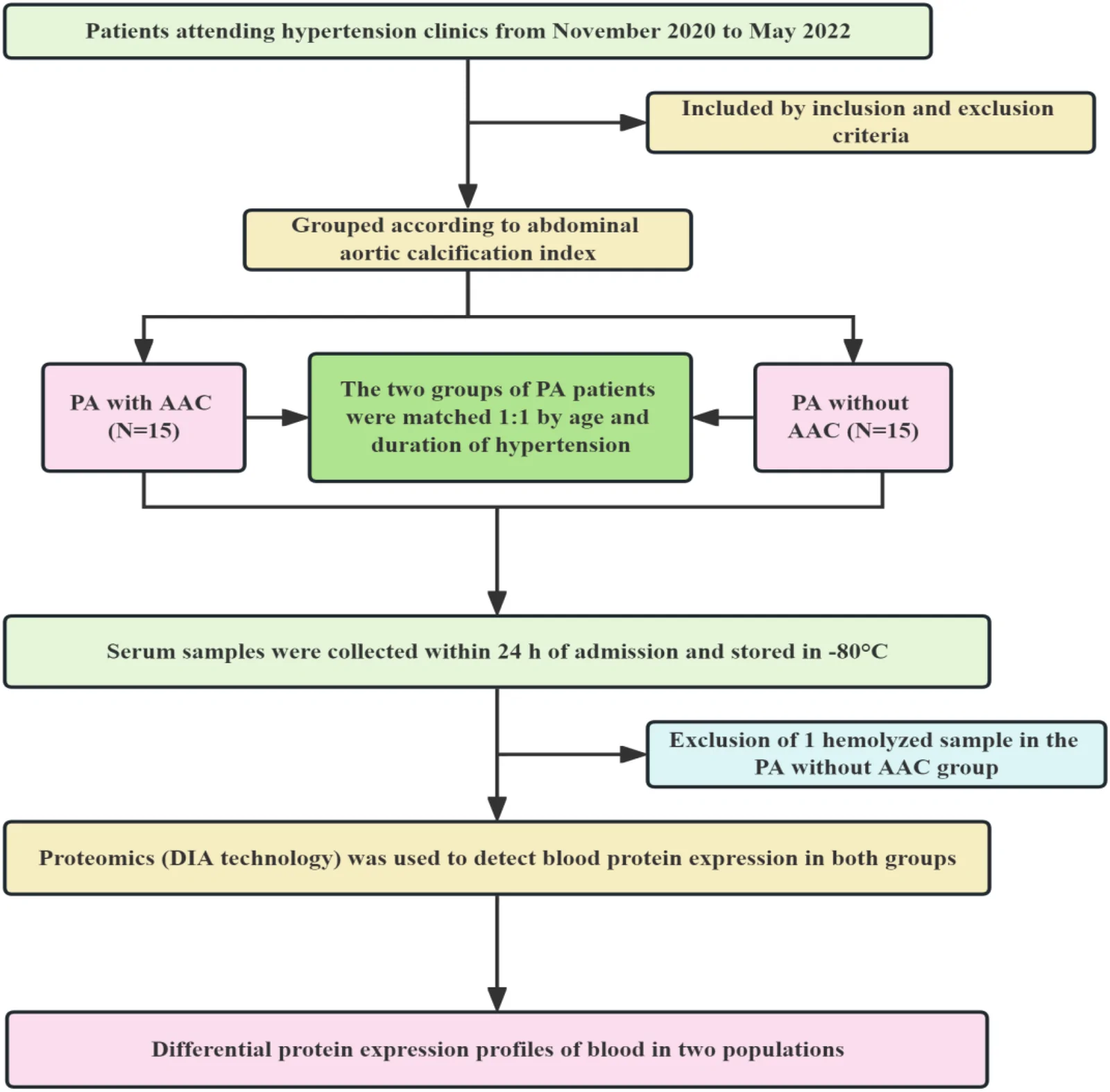
Flowchart of patient enrollment, grouping, and sample processing. Patients attending hypertension clinics from November 2020 to May 2022 were screened by inclusion and exclusion criteria, then grouped according to abdominal aortic calcification index. Two groups of primary aldosteronism (PA) patients (PA with abdominal aortic calcification [AAC], N = 15; PA without AAC, N = 15) were matched 1:1 by age and duration of hypertension. Serum samples were collected within 24 h of admission and stored at -80 °C. One hemolyzed sample was excluded from the PA without AAC group. Data-independent acquisition (DIA) proteomics was applied to detect blood protein expression in both groups, and differential protein expression profiles were finally obtained. PA, primary aldosteronism; AAC, abdominal aortic calcification; DIA, data-independent acquisition.

### 2.2 Diagnosis of primary aldosteronism

PA was screened using an aldosterone-to-renin ratio (ARR) ≥20 (ng/dL)/(ng/mL/h) and plasma aldosterone concentration (PAC) ≥12 ng/dL. Confirmatory diagnosis was performed via the saline infusion test (SIT) according to Endocrine Society guidelines (20, 21). Briefly, patients remained seated for 30 minutes before and during infusion of 2 L of 0.9% saline over 4 hours. Post-infusion PAC >10 ng/dL confirmed PA, while PAC 5–10 ng/dL was considered indeterminate, and PAC <5 ng/dL excluded PA. All antihypertensive medications, including angiotensin-converting enzyme inhibitors, angiotensin receptor blockers, dihydropyridine calcium antagonists and β-blockers, were discontinued for at least 4 weeks, and diuretics and mineralocorticoid receptor antagonists for at least 6 weeks prior to ARR measurement. If medication withdrawal was not feasible, patients were switched to α-blockers or extended-release calcium channel blockers. Calcium and vitamin D supplements were withheld for 4–6 weeks before testing.

### 2.3 Clinical data collection

General information, medical history, and personal history were recorded, including gender, age, height, weight, admission blood pressure, and hypertension duration. Fasting venous blood samples were collected for the measurement of serum lipids, creatinine, potassium, calcium, PAC, and plasma renin activity (PRA) using matched commercial kits on an automatic biochemical analyzer. All patients underwent adrenal computed tomography (CT). Blood pressure was measured under standardized protocols. Subjects rested quietly for at least 5 min and avoided coffee, smoking and urination 30 min before measurement. Bilateral upper arm blood pressure was detected three times at 2-min intervals, and the average value was adopted if the difference between repeated readings was less than 5 mmHg.

### 2.4 Measurement of abdominal aortic calcification index

Abdominal aortic calcification index (ACI) was assessed via adrenal thin-slice CT (3 mm thickness) performed from 10 mm above to 10 mm below the origin of the left renal artery. AAC was defined as a lesion ≥1 mm² with density ≥130 Hounsfield units. Each aortic cross-section was divided into 12 radial segments. ACI was calculated as 100 × (total calcified segments/120). ACI >0 indicated the presence of AAC, while ACI = 0 indicated no AAC (21, 22) (**Figure 2**). All CT images were independently evaluated by two radiologists blinded to patient grouping, and intraclass correlation coefficient (ICC) was calculated to assess inter-rater consistency of aortic calcification scoring.

**Figure 2:**
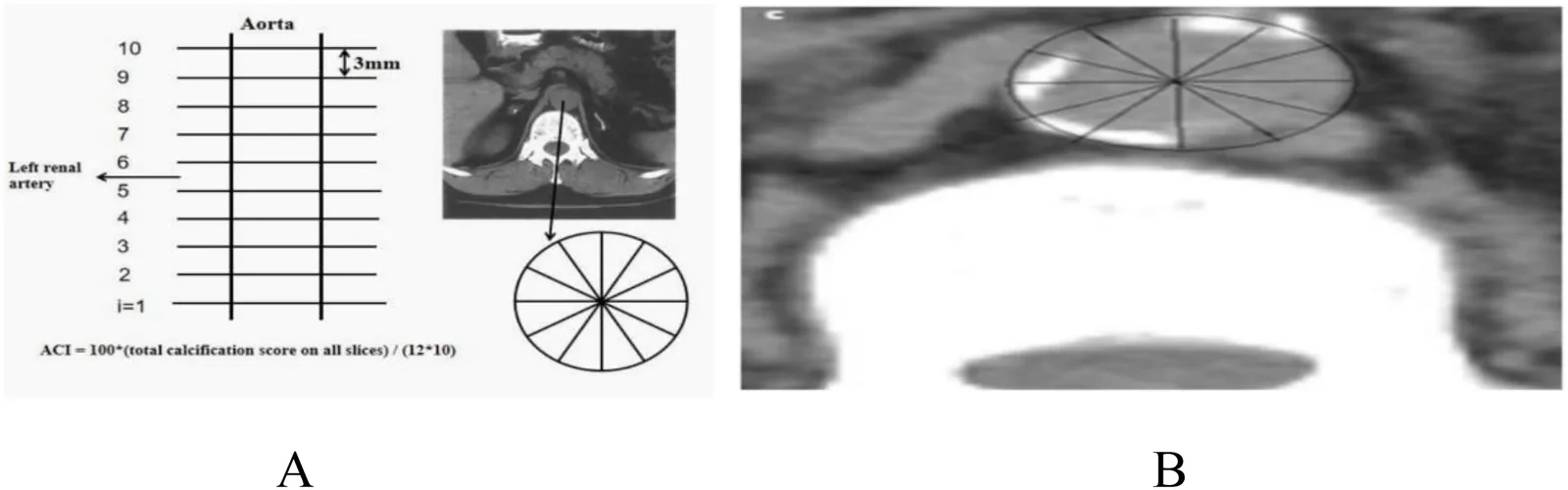
Schematic illustration of abdominal aortic calcification index (ACI) assessment (21, 22). **(A)** Diagram of the slice segmentation and sector division for ACI calculation. **(B)** Representative CT cross-sectional image of abdominal aorta with calcified sectors. Every participant received thin-section adrenal CT examination. Scanning began at the left renal artery plane and contained ten sequential slices with a thickness of 3 mm each. Tissue regions reaching 1 mm² or larger with a density reading above 130 Hounsfield units were judged as calcified lesions. For every scanned slice, the transverse section of the abdominal aorta was split radially into 12 equal sectors, followed by counting calcified sectors on each slice. The formula used to calculate ACI: ACI = 100 × total calcified sectors from all slices/(12 × 10). ACI values represent the severity of abdominal aortic calcification (AAC); an ACI value greater than zero confirms the existence of AAC, whereas an ACI value of zero signifies no vascular calcification observed. AAC, abdominal aortic calcification; ACI, abdominal aortic calcification index; CT, computed tomography.

### 2.5 DIA-based quantitative proteomic profiling

#### 2.5.1 Serum pretreatment and total protein extraction

Venous blood was collected within 24 h of admission, centrifuged at 3000 × g for 10 min at 4 °C, and serum aliquots were stored long-term in a −80 °C ultra-low freezer (Xueke, Model DW-86L338). For each sample, 40 μL serum was diluted 10-fold with binding buffer and loaded onto Millipore spin columns to deplete high-abundance albumin and immunoglobulins. Depletion efficiency was verified by SDS-PAGE electrophoresis. After sequential washing steps, target protein eluates were vacuum-dried in a Taicang Kejiao ZLS-1 concentrator and reconstituted in Beyotime RIPA lysis buffer supplemented with 1 mM Roche protease and phosphatase inhibitor cocktails.

Total protein concentration was determined via Thermo Scientific BCA assay, with absorbance read at 562 nm on an Implen NanoPhotometer NP80. Equal protein mass from each sample was subjected to denaturation, alkylation and tryptic digestion: samples were incubated with 25 mM DTT (Damas-beta) at 55 °C for 30 min, followed by 15-min dark alkylation with 100 mM iodoacetamide (Sangon Biotech) at room temperature. Proteins were precipitated overnight at −20 °C using 6 volumes of chromatographic-grade acetone (CNW Technologies). Air-dried protein pellets were dissolved in 50 mM ammonium bicarbonate (Yuanye Bio) and digested overnight at 37 °C with TPCK-treated trypsin (HLS, Hualishi) at a protein:enzyme mass ratio of 50:1. Digestion was terminated by adjusting pH to ~3 with formic acid (Thermo Fisher).

### 2.6 Peptide desalting and prefractionation

Digested peptide mixtures were desalted on Thermo Fisher 96-well C18 SPE plates and vacuum-dried. Biognosys iRT calibration peptides were mixed with sample peptides at a 1:10 volume ratio for retention time normalization. High-pH reversed-phase prefractionation was performed on an Agilent 1100 HPLC equipped with a Zorbax Extend-C18 column (2.1 × 150 mm, 5 μm). Mobile phase A consisted of 2% acetonitrile in water, mobile phase B of 90% acetonitrile in water; both phases were adjusted to pH 10 with ammonia water at a flow rate of 250 μL/min. The linear gradient was set as: 0–10 min 2% B; 10–37 min linear ramp to 20% B; 37–48 min linear ramp to 40% B; 48–58 min hold at 90% B; 58–63 min re-equilibrate to 2% B. Eluted fractions were collected every 1 min, pooled and vacuum-dried for nano-LC-MS analysis. Pooled quality-control (QC) samples were injected periodically throughout the LC-MS acquisition workflow to monitor instrument performance and quantitative stability. No technical replicate injections were performed for individual biological serum samples.

### 2.7 Nano-LC-DIA mass spectrometry

Peptide separation was conducted on a Thermo Fisher Easy-nLC 1200 system coupled to a Q Exactive Plus Orbitrap mass spectrometer under standard DIA acquisition parameters: spray voltage 2.2 kV, ion transfer tube temperature 320 °C; full MS scan range m/z 350–1400, resolution 120,000 at m/z 200, AGC target 3 × 10^6^, maximum injection time 100 ms; DIA isolation window width 20 m/z, MS/MS resolution 30,000, AGC target 1 × 10^6^, maximum injection time 50 ms. Mobile phases were 0.1% formic acid aqueous solution (A) and 0.1% formic acid in acetonitrile (B), delivered at a constant flow rate of 300 nL/min. After completing GO functional annotation, KEGG pathway enrichment and protein-protein interaction (PPI) network analyses of all differentially expressed proteins, we chose tenascin-X (TNX, encoded by the *TNXB* gene) as the key candidate for further cellular and animal experimental validation. To our knowledge, abnormal TNX expression has not been previously reported among PA patients suffering from AAC, and no prior works have explored this correlation so far. Other highly connected hub proteins in the interaction network have been extensively characterized in former vascular calcification researches. Focusing on TNX improves the novelty of the present work.

### 2.8 Cell culture and *in vitro* intervention assays

#### 2.8.1 Cell lines, reagents and instruments

MOVAS mouse aortic vascular smooth muscle cells were purchased from Saibikang Biotechnology, authenticated via STR profiling and confirmed mycoplasma-negative before use. Complete culture medium consisted of high-glucose DMEM supplemented with 10% fetal bovine serum (Excell, FND500) and 1% penicillin-streptomycin mixture (Gibco, 15070-063), incubated at 37 °C with 5% CO_2_ and saturated humidity; proprietary MOVAS special medium (iCell-m088-001b, Saibikang) was adopted for routine maintenance.

Key experimental reagents: 0.25% Trypsin-EDTA (Gibco, 25200-056), ZSGB-BIO PBS powder, DMSO (Sigma D2650), aldosterone (MCE HY-113313), rabbit polyclonal anti- TNX (13595-1-AP). Consumables included 15 mL centrifuge tubes (Excell CS015-0001), 24-well culture plates (Corning 3524) and 25 cm² cell culture flasks (Corning 430639).

Major instruments: Hikon Smart Cell HF-90 CO_2_ incubator, HF1200LC biosafety cabinet, Jinghong DK-80 low-speed centrifuge and TGL-16GB water bath, Meiling −20 °C BCD-249LCK and −80 °C DW-HL388 freezers, Eppendorf Research plus pipettes, Jinfeng YDS-50 liquid nitrogen tank, Nikon Eclipse TS100-F inverted fluorescence microscope, Qinxiang Chemiscope 3000 chemiluminescence imager, Bio-Rad Mini-PROTEAN Tetra western blot transfer system.

#### 2.8.2 Routine cell manipulation

##### 2.8.2.1 Cell resuscitation

Frozen cell vials were rapidly thawed in a 37 °C water bath, transferred into 15 mL tubes preloaded with 9 mL complete medium, centrifuged at 1000 rpm for 5 min, supernatant discarded, and cell pellets resuspended and seeded into culture flasks for standard incubation.

##### 2.8.2.2 Cell passaging

Residual medium was aspirated, cell monolayers washed 3 times with sterile PBS, 1 mL trypsin added to cover adherent cells, and flasks incubated for 1 min to achieve digestion. Digestion was terminated with an equal volume of complete medium; cell suspension was collected, centrifuged at 1000 rpm for 5 min, and pellets resuspended in fresh medium for subculture at appropriate density.

##### 2.8.2.3 Cell cryopreservation

Cryopreservation medium was freshly prepared (400 μL FBS + 100 μL DMSO per cryovial). Cells reaching 90% confluence were digested, centrifuged and resuspended in 500 μL complete medium, transferred into prepared cryovials. Programmed cooling was applied: 4 °C for 20 min, −20 °C for 30 min, overnight storage at −80 °C, followed by long-term preservation in liquid nitrogen.

#### 2.8.3 Aldosterone intervention and Western blot detection of TNX

Aldosterone powder was dissolved in DMSO to prepare stock solution. The blank control group was cultured with medium containing an equal volume of DMSO solvent to eliminate solvent interference, while the intervention group was treated with 1 μM aldosterone continuously for 6 days.

After 6 days of intervention, total cellular protein was extracted from both groups for western blot detection of TNX expression levels. Briefly, equal amounts of total protein were separated by 12% SDS-PAGE (80 V then 100 V electrophoresis for 90 min), transferred to 0.45 μm PVDF membranes via 100 V wet transfer (80 min for TNX/β-actin), blocked with 5% skim milk powder, and incubated with primary antibodies targeting TNX (1:500) overnight at 4 °C. After PBS washing, HRP-conjugated secondary antibody was incubated at room temperature, and β-actin was used as the internal reference protein. Target band signals were captured via chemiluminescence imaging equipment for subsequent grayscale quantitative analysis. Full western blot operating procedures were integrated within this manuscript rather than relying on external independent documents.

### 2.9 *In vivo* validation

#### 2.9.1 *In vivo* validation: immunohistochemistry for TNX in mouse aortic tissues

##### 2.9.1.1 Animal model establishment and tissue preparation

Eight 4-month-old (16-week-old) male C57BL/6J mice with body weights ranging from 28 to 33 g were randomly allocated into control and aldosterone intervention groups (n=4 for each group). Subcutaneous osmotic mini-pumps were used to deliver aldosterone continuously for 28 consecutive days to maintain a daily systemic dosage of 500 μg/kg body weight. Aldosterone powder was dissolved in sterile 0.9% saline as vehicle, and identical mini-pumps loaded with pure saline were implanted in control mice. All mice were housed under standardized laboratory conditions: a 12 h light/dark cycle, constant temperature at 22–24 °C, and unlimited access to standard rodent chow and sterile drinking water.

Continuous aldosterone delivery was achieved via subcutaneous osmotic mini-pump implantation for 28 consecutive days; the control group received identical mini-pumps loaded with sterile normal saline vehicle. After 28 days of intervention, all mice were sacrificed under deep anesthesia, whole aortic tissues dissected, fixed and processed into FFPE paraffin blocks using TSJ-II dehydrator and BMJ-III embedding equipment. Continuous 4 μm serial slices were cut on a Leica RM2016 microtome with Feather R35 disposable blades, mounted on Meiweid P105–2001 glass slides and dried in a Jinghong DHG-9148A drying oven. Labserv BC004 hydrophobic pens were used to draw isolation boundaries around tissue sections to prevent reagent loss during incubation.

##### 2.9.1.2 Stepwise immunohistochemistry staining protocol

Slides underwent triple xylene dewaxing (15 min per batch), gradient ethanol rehydration (100%, 95%, 85%, 75%, 5 min each), followed by distilled water rinsing. Antigen retrieval was performed with 1:1000 diluted citrate buffer (Servicebio GA2307051) heated in a Thermo A8040001 retrieval instrument for 20 min, slides cooled naturally and washed 3 × 5 min with PBS (Servicebio G0002-2L) on a Chenjie HY-5 horizontal shaker.

Endogenous peroxidase activity was blocked by 25-min dark incubation with 0.5% H_2_O_2_ (Servicebio G1204), followed by repeated PBS washes. Non-specific binding was blocked with 3% BSA blocking solution (Servicebio GC305010) at room temperature for 20 min. Negative control slices incubated without primary antibody were set up to exclude non-specific background staining. Rabbit anti-TNX primary antibody (Proteintech 13595-1-AP, 1:100 dilution) was evenly spread on sections, incubated overnight at 4 °C in a humid box, washed 3 times with PBS, then incubated with HRP-labeled goat anti-rabbit secondary antibody (Servicebio GB22303, 1:100) at 37 °C for 30 min.

Freshly prepared DAB working solution (Zhongshan Golden Bridge ZL1-9018, diluted 1:20) was applied for chromogenic reaction under real-time light microscopy observation; staining was terminated with distilled water once distinct brown positive signals appeared. Nuclei were counterstained with hematoxylin (Bailingwei LM10N13) for 3 min, differentiated with hematoxylin differentiation liquid (Servicebio G1039), blued with bluing reagent (Servicebio G1040), subjected to gradient ethanol dehydration and xylene clearing, and finally mounted with neutral mounting medium and coverslips.

### 2.10 Statistical analysis

Statistical processing of all datasets in this work was implemented on SPSS 19.0, SPSS 21.0 and R 4.6.1 software suites. Raw mass spectrometry files generated from DIA serum proteomics were processed using Biognosys Spectronaut Pulsar v15.3.210906.50606 to build spectral libraries, quantify relative protein abundance and conduct protein identification; the false discovery rate (FDR) threshold for credible protein identification was fixed below 0.01.

Prior to any intergroup comparison of clinical continuous indicators, cellular readouts and mouse aortic quantitative data, normality assessment was performed for each dataset. Normally distributed variables were presented as mean ± standard deviation and compared via independent two-sample t-tests, while non-normal skewed data were reported as median with interquartile ranges and analyzed with the Mann-Whitney U test.

For differential protein screening in DIA proteomics, proteins with fold change (FC) ≥ 1.2 or ≤ 0.83 together with raw *P* < 0.05 were identified as DEPs, consistent with analytical pipelines reported in previous high-impact omics studies (23, 24). FDR-based multiple testing correction was not adopted in the present research; alternatively, a strict fold-change cutoff was applied to minimize false positive hits, an analytical strategy widely utilized in small-scale serum proteomic cohorts. The robustness of our screening outcomes was further validated by separate *in vitro* vascular smooth cell assays and *in vivo* mouse aortic tissue measurements, which could partially mitigate the risk of false-positive hits derived from omics-based screening.

Three independent biological replicates were prepared for all cellular functional experiments. For *in vivo* validation, male C57BL/6J mice were evenly assigned to control and aldosterone treatment groups (n = 4 per group). All quantitative analysis of immunohistochemical staining was completed by researchers blinded to animal grouping using Indica Labs Halo 101-WL-HALO analytical software.

All image acquisition and quantitative analysis were completed by researchers blinded to mouse group allocation to avoid subjective bias. Three random 40× magnification fields were captured from each aortic section using a Motic BA400Digital trinocular digital microscope. The proportion of DAB-positive stained area was quantified via Indica Labs Halo 101-WL-HALO analysis software. We adopted a two-tailed threshold of *P* < 0.05 to judge whether intergroup comparisons yielded statistically meaningful differences. Instead of splitting P values into rigid two-stage or multi-level significance grades, we treated them as continuous metrics that reflect the magnitude of statistical evidence (25). As stated in the official ASA statement on *p*-values, a single fixed threshold cannot mechanically define research conclusions; there is no universally recognized standard range for “borderline significance”, *P* values near 0.05 only provide weak numerical clues rather than sufficient evidence to confirm intergroup differences, and *P* ≥ 0.05 cannot be interpreted as the absence of biological effects, which is usually attributable to limited sample size and insufficient statistical power (25). Following standard reporting practices widely accepted across biomedical studies (26), as well as descriptive conventions used in numerous high-ranking academic journals (27), we merely state directional numerical shifts for all measured markers with *P* ≥ 0.05 in this mouse model. Any mild between-group numerical gaps observed are explained by the small animal cohort size and inadequate statistical power.

## 3 Results

### 3.1 Differential protein screening results in patients with primary aldosteronism combined with and without abdominal aortic calcification

#### 3.1.1 Baseline characteristics

As shown in **Table 1**, this study included a total of 29 serum samples, with 15 cases in the PA with AAC group and 14 cases in the PA without AAC group. The mean age of participants in the PA with AAC group was 52.40 ± 3.70 years, with mean admission systolic blood pressure of 139.47 ± 13.39 mmHg and median diastolic blood pressure of 82.00 (80.00, 98.00) mmHg, and the duration of hypertension in this group was 7.60 ± 2.06 years. In the PA without AAC group, the mean age was 52.14 ± 4.26 years, with mean admission systolic blood pressure of 142.50 ± 20.18 mmHg and median diastolic blood pressure of 85.50 (80.00, 92.75) mmHg, and the duration of hypertension was 7.79 ± 2.05 years. No significant inter-group differences were observed in age, gender, blood pressure, BMI, hypertension duration, PAC, PRA, ARR, serum potassium, calcium and creatinine (all P>0.05). Significant differences in serum triglyceride and HDL-C were noted between groups. As expected, ACI values differed markedly between AAC and non-AAC groups (P < 0.001)(**Table 1**).

**Table 1:** Baseline clinical characteristics of PA patients with and without AAC.

Characteristic	Total	PA with AAC	PA without AAC	*P*
N	29	15	14	—
Age	52.28 ± 3.91	52.40 ± 3.70	52.14 ± 4.26	0.86
Gender				0.92
Male	6(20.70%)	3(20.00%)	3(21.40%)	
Female	23(79.30%)	12(80.00%)	11(78.60%)	
ACI	2.50 (0.00,4.98)	4.17 (3.30,8.30)	0	<0.001
Systolic blood pressure (mmHg)	140.93 ± 16.77	139.47 ± 13.39	142.50 ± 20.18	0.64
Diastolic blood pressure (mmHg)	83.00 (80.00,95.00)	82.00 (80.00,98.00)	85.50 (80.00,92.75)	0.84
Body mass index (kg/m2)	25.26 (22.85,26.72)	25.39 (23.60,28.87)	24.46 (22.61,25.58)	0.35
Duration of hypertension (years)	7.69 ± 2.02	7.60 ± 2.06	7.79 ± 2.05	0.81
PAC (ng/dl)	11.75 (8.84,14.31)	11.75 (9.26,14.15)	11.22 (8.80,15.57)	0.86
PRA (ng/ml/h)	0.57 (0.36,0.74)	0.60 (0.42,0.86)	0.40 (0.30,0.62)	0.08
ARR	34.43 (22.73,61.56)	25.86 (20.74,40.28)	52.61 (23.42,67.30)	0.10
Pre-SIT PAC (ng/dl)	19.15 (15.15,23.02)	19.97 (14.74,24.17)	18.01 (15.58,22.92)	1.00
K+(mmol/L)	3.59 ± 0.22	3.55 ± 0.21	3.63 ± 0.24	0.34
Ca+(mmol/L)	2.19 ± 0.09	2.19 ± 0.10	2.18 ± 0.09	0.65
TG (mmol/L)	1.12 (0.83,1.49)	1.37 (1.12,1.95)	0.93 (0.77,1.11)	0.004
LDL-C(mmol/L)	2.28 ± 0.89	2.23 ± 0.88	2.34 ± 0.93	0.75
TC (mmol/L)	4.07 ± 0.97	4.00 ± 1.08	4.15 ± 0.88	0.69
HDL-C(mmol/L)	1.23 ± 0.26	1.12 ± 0.27	1.35 ± 0.21	0.02
Creatinine (umol/L)	60.91 ± 9.06	60.70 ± 10.73	61.14 ± 7.28	0.90

PAC, Plasma aldosterone concentration; PRA, Plasma renin activity; ARR, Aldosterone-to-renin ratio; TG, Triglycerides; LDL-C, Low-density lipoprotein cholesterol; TC, Total cholesterol; HDL-C, High-density lipoprotein cholesterol; PA, Primary aldosteronism; AAC, Abdominal aortic calcification. Data conforming to normal distribution were shown as mean ± SD and analyzed by independent t-test; non-normal data were expressed as median (interquartile range) and assessed with Mann-Whitney U test.

### 3.2 Results of differential expression analysis by serum protein mass spectrometry

A total of 741 plausible proteins were detected in this study using DIA mass spectrometry. Among them, 38 differentially expressed proteins were identified, including 6 proteins with upregulated expression and 32 proteins with downregulated expression. The volcano plot and heat map of these differentially expressed proteins are shown in **Figure 3** and **4**. Detailed information and expression fold for the 38 proteins are provided in **Supplementary Table S1**. (**Figure 3**, **4**) (**Supplementary Table S1**).

**Figure 3:**
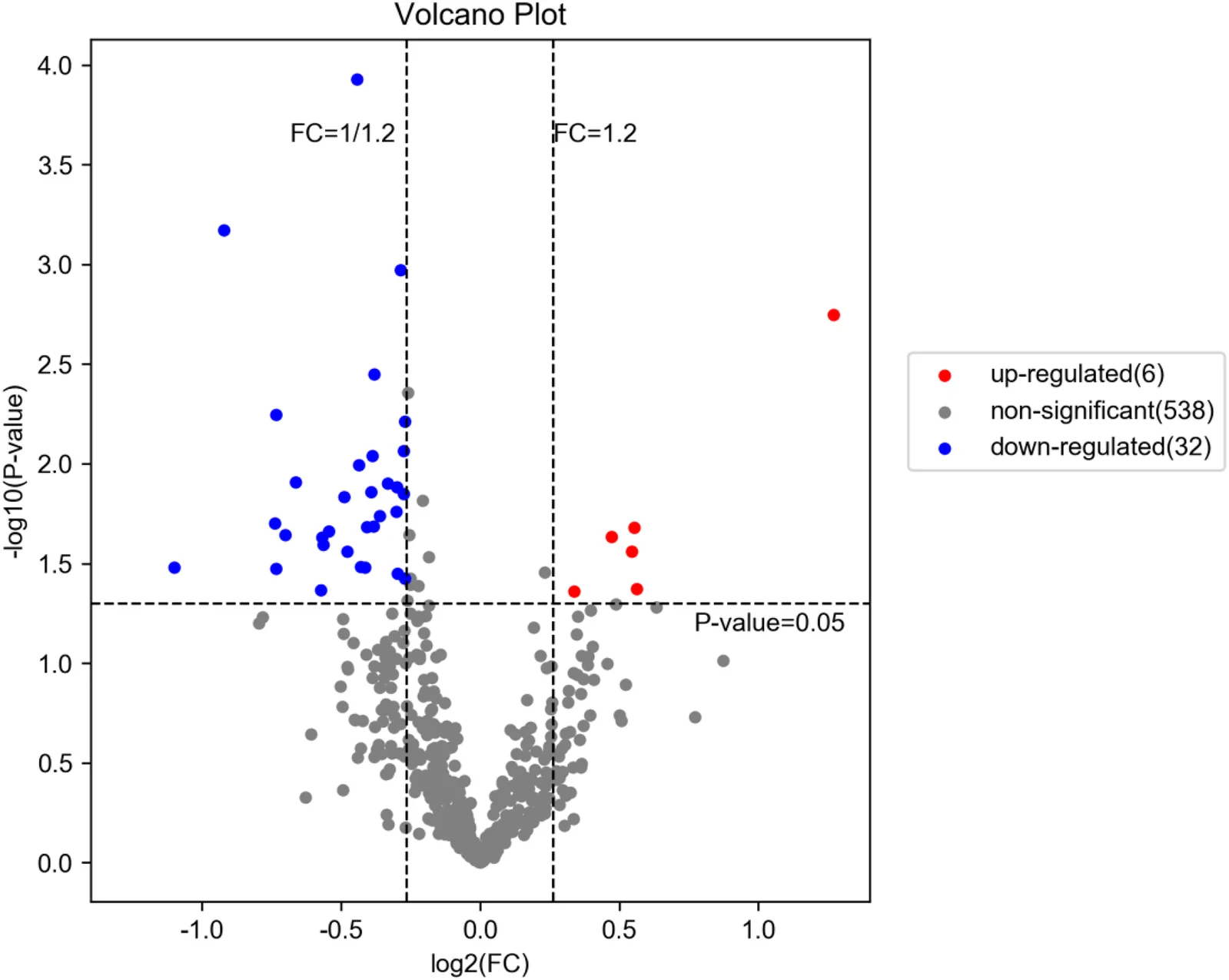
Volcano plot of serum differentially expressed proteins (DEPs) comparing PA patients with AAC (n = 15) and PA patients without AAC (n = 14). X-axis = log_2_(fold change, FC); Y-axis = −log_10_(*P*-value). Red dots: upregulated DEPs (FC ≥ 1.2, *P* < 0.05, n=6); blue dots: downregulated DEPs (FC ≤ 0.83, *P* < 0.05, n=32); gray dots: non-significant proteins. PA, primary aldosteronism; AAC, abdominal aortic calcification; DEP, differentially expressed protein; FC, fold change. Of the total 741 detected proteins, 576 proteins passing missing-value and signal-to-noise pre-filtering were used for differential-expression analysis and volcano plot generation.

**Figure 4:**
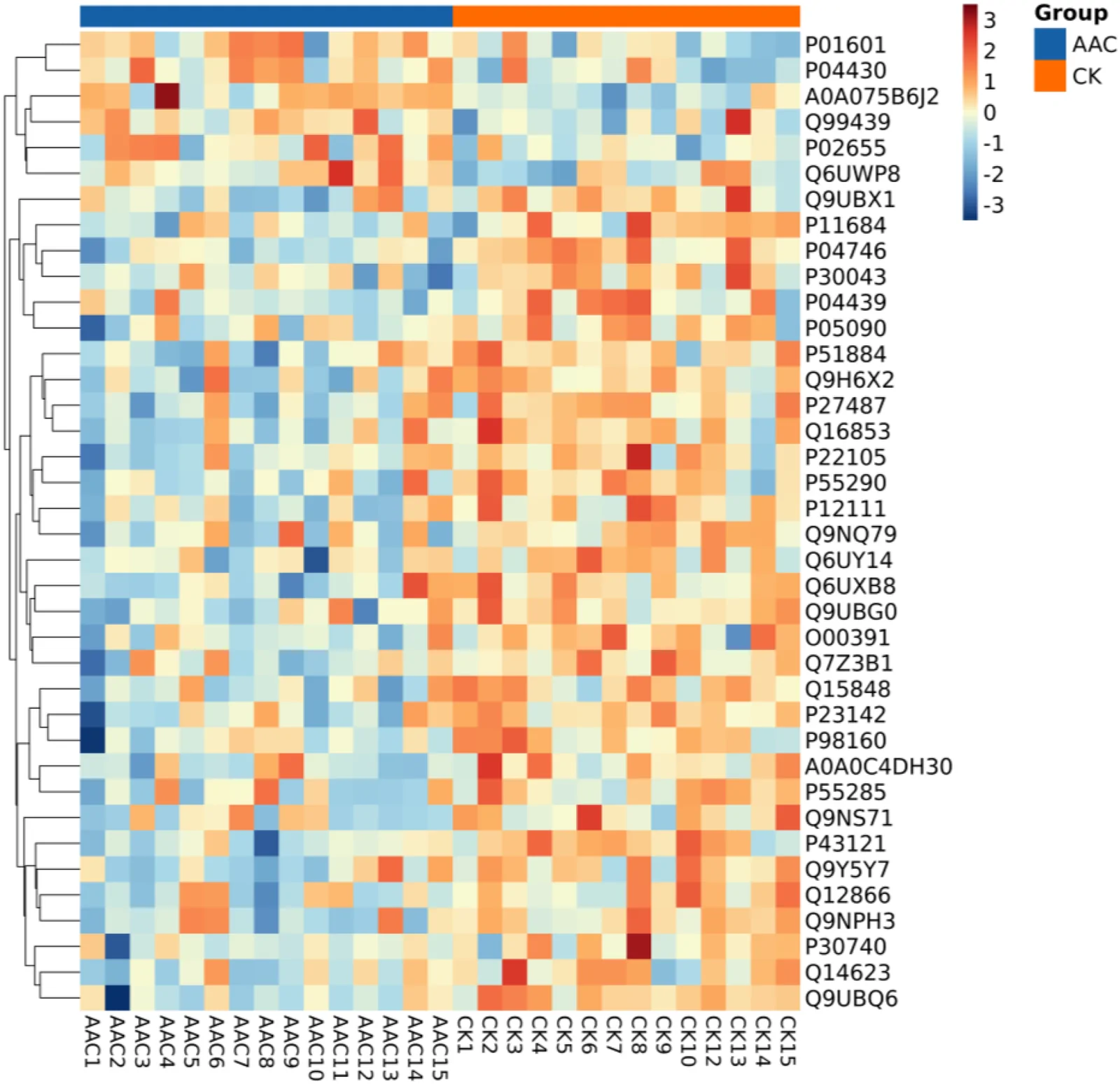
Hierarchical clustering heatmap of all DEPs identified by comparing PA patients with AAC and PA patients without AAC. Blue top bar = PA with AAC group (n=15); orange top bar = PA without AAC control group (CK, n=14). Color gradient: blue = low protein expression, red = high protein expression. PA, primary aldosteronism; AAC, abdominal aortic calcification; CK, control group; DEP, differentially expressed protein.

### 3.3 GO functional annotation and enrichment analysis

The results of the GO enrichment analysis, including Biological Process, Cellular Component, and Molecular Function, for differentially expressed proteins in patients with PA and AAC versus those without AAC, are shown in **Figure 5**.

**Figure 5:**
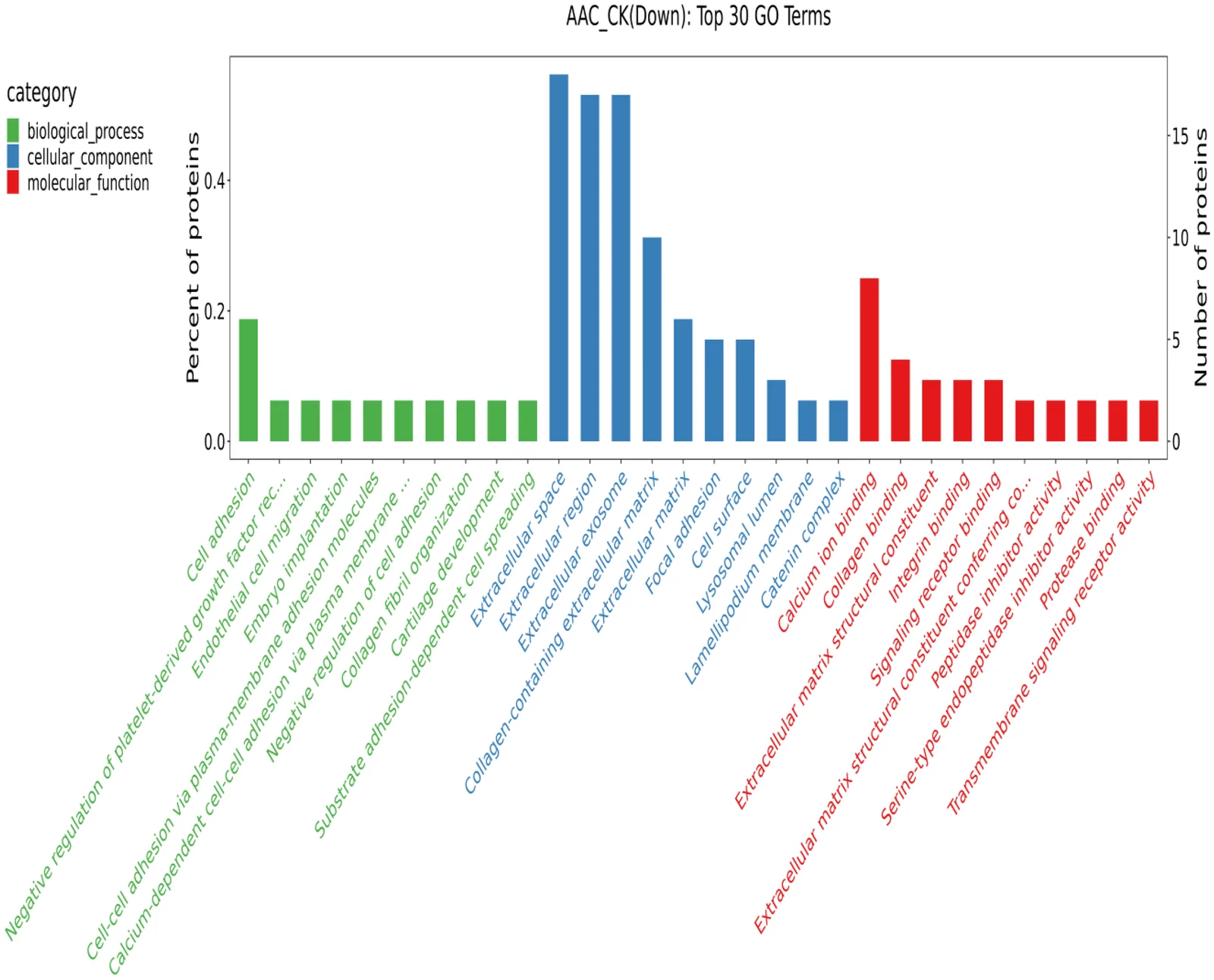
Top 30 significantly enriched Gene Ontology (GO) terms of downregulated differentially expressed proteins (DEPs) in PA patients with AAC compared to PA patients without AAC. Green bars correspond to biological process terms, blue bars to cellular component terms, and red bars to molecular function terms. The left vertical axis represents the proportion of enriched proteins, while the right vertical axis indicates the total number of enriched proteins for each term. DEP, differentially expressed protein; GO, Gene Ontology; PA, primary aldosteronism; AAC, abdominal aortic calcification.

The GO analysis results revealed that differentially expressed proteins (DEPs) in Biological Processes (green) were primarily associated with cell adhesion and immune response. In Molecular Functions (red), DEPs were mainly linked to calcium binding, collagen binding, and antigen binding. In Cellular Components (blue), DEPs were predominantly found in extracellular regions, cellular exosomes, and extracellular organelles (**Figure 5**).

### 3.4 KEGG pathway annotation and enrichment analysis

As shown in **Figure 6**, KEGG pathway analysis revealed that differentially expressed proteins (DEPs) were primarily involved in extracellular matrix receptor interactions, mucins in cancer, adhesion plaques, and protein digestion and absorption. The pathways with the highest abundance of DEPs were related to extracellular matrix receptor interactions (COL6A3, HSPG2, TNX) and mucins in cancer (LUM, HSPG2, IHH). The degree of enrichment significantly differed between the two groups (**Figure 6**).

**Figure 6:**
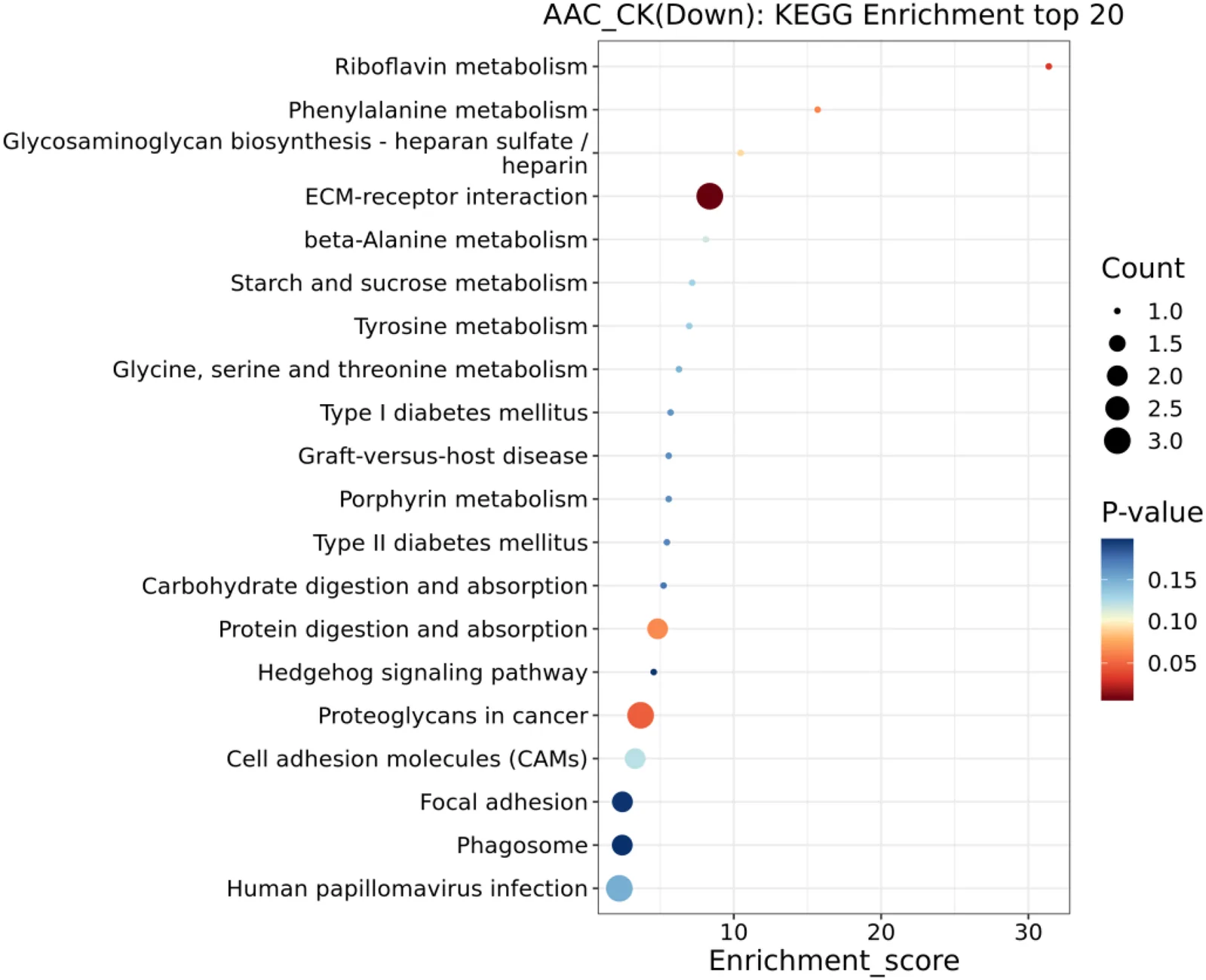
Top 20 KEGG enrichment pathways for DEPs downregulated in PA patients with AAC versus PA patients without AAC. Dot size corresponds to the count of enriched proteins; dot color gradient represents *P*-value (red = smaller *P*-value). X-axis = enrichment score. DEP, differentially expressed protein; KEGG, Kyoto Encyclopedia of Genes and Genomes; ECM, extracellular matrix.

### 3.5 Protein-protein interaction network analysis of differential proteins

As shown in **Figure 7**, the PPI network was built using all 38 DEPs in the STRING database, and the top 25 nodes ranked by connectivity degree were visualized. Notably, these high-connectivity nodes were all down-regulated, with LUM showing the highest node degree (**Figure 7**).

**Figure 7:**
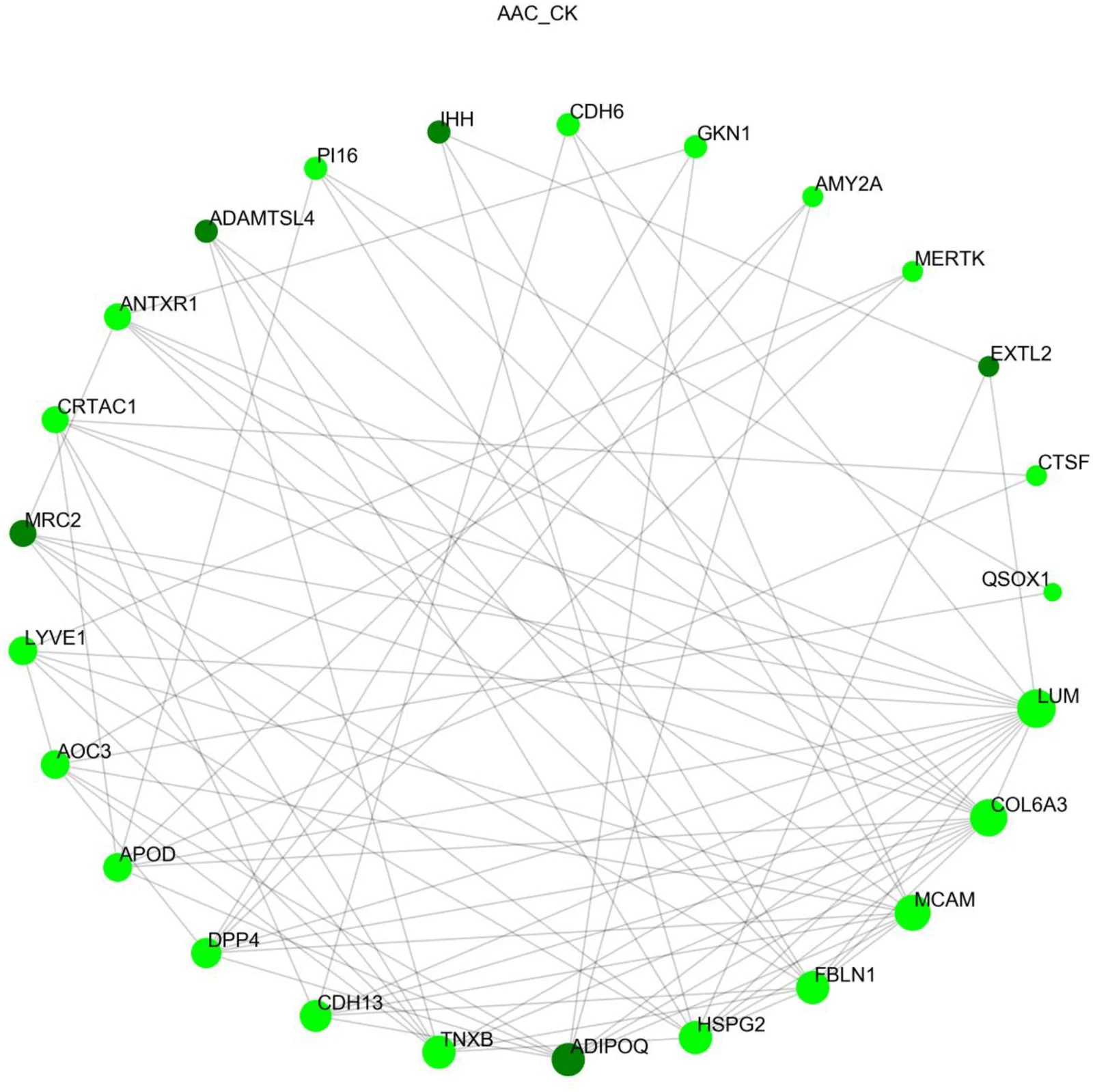
Protein-protein interaction (PPI) network of differentially expressed proteins (DEPs). All 38 DEPs were imported into STRING. The top 25 nodes with highest connectivity are shown; all of these highly connected nodes were down-regulated. TNXB is highlighted in dark-green. PPI, protein-protein interaction; DEP, differentially expressed protein.

Notably, the observed downregulation of TNX in PA patients with AAC has not been previously reported. Though LUM exhibited the highest node degree in PPI network, its roles in common vascular calcification have been extensively documented with limited novelty in PA-specific settings, so we prioritized TNX for multi-level validation. Therefore, this protein was selected for further validation at both the cellular and animal levels.

### 3.6 Validation by cell-based experiments

As summarized in **Table 2**, the expression level of TNX in vascular smooth muscle cells was significantly reduced in the aldosterone-treated group (0.302 ± 0.038) compared with the control group (0.608 ± 0.022), with a statistically significant difference (*P* < 0.001). Consistent with the quantitative results, the representative figure also demonstrates markedly lower TNX expression in the aldosterone group relative to controls. (**Table 2**) (**Figure 8**).

**Table 2:** Quantitative analysis of TNX expression in vascular smooth muscle cells.

Protein	Control	Aldosterone group	*P*-value
TNX	0.608 ± 0.022	0.302 ± 0.038	<0.001

Data represent mean ± SD of Western blot gray values; n = 3 biological replicates per group; Data conforming to normal distribution were shown as mean ± SD and analyzed by independent t-test; non-normal data were expressed as median (interquartile range) and assessed with Mann-Whitney U test.

**Figure 8:**
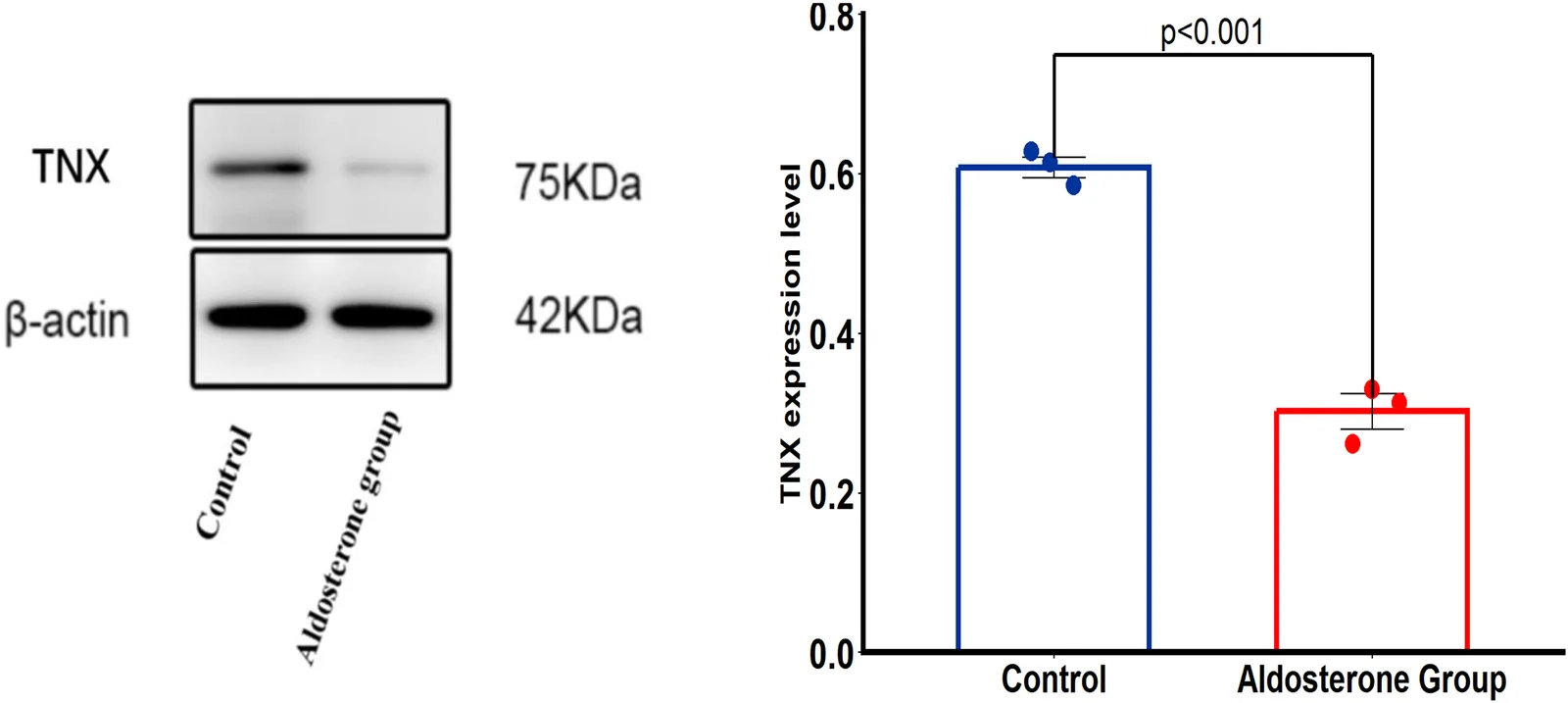
Representative western blot bands and quantitative scatter-bar graph for TNX expression in vascular smooth muscle cells cultured with or without 1 μM aldosterone for six days (n = 3 biological replicates per group). Intergroup difference *P* < 0.001. Bar plots present mean ± SD, and scattered dots stand for the expression level of each individual biological replicate. VSMC, vascular smooth muscle cell;.

### 3.7 Mouse aortic tissue validation results

We selected aortic tissues from aldosterone-intervened mice as well as control mice (4 mice in each group) for the detection of the relevant proteins, and found that there was a trend toward a decrease in TNX expression in the aldosterone-intervened group (*P* = 0.054). This trend provides suggestive directional clues for our previous findings; this concordant trend requires further confirmation in a larger-sample animal group. (**Table 3**) (**Figure 9**, **10**).

**Table 3:** Quantitative analysis of TNX immunohistochemical positive area in mouse aortic tissue.

Protein	Control	Aldosterone group	*P*-value
TNX	5.436 ± 0.855	2.832 ± 1.783	0.054

Data represent mean ± SD of immunohistochemical positive area; n = 4 male C57BL/6J mice per group; The intergroup *P* value was 0.054, showing a borderline decreasing trend without statistical significance; Data conforming to normal distribution were shown as mean ± SD and analyzed by independent t-test; non-normal data were expressed as median (interquartile range) and assessed with Mann-Whitney U test.

**Figure 9:**
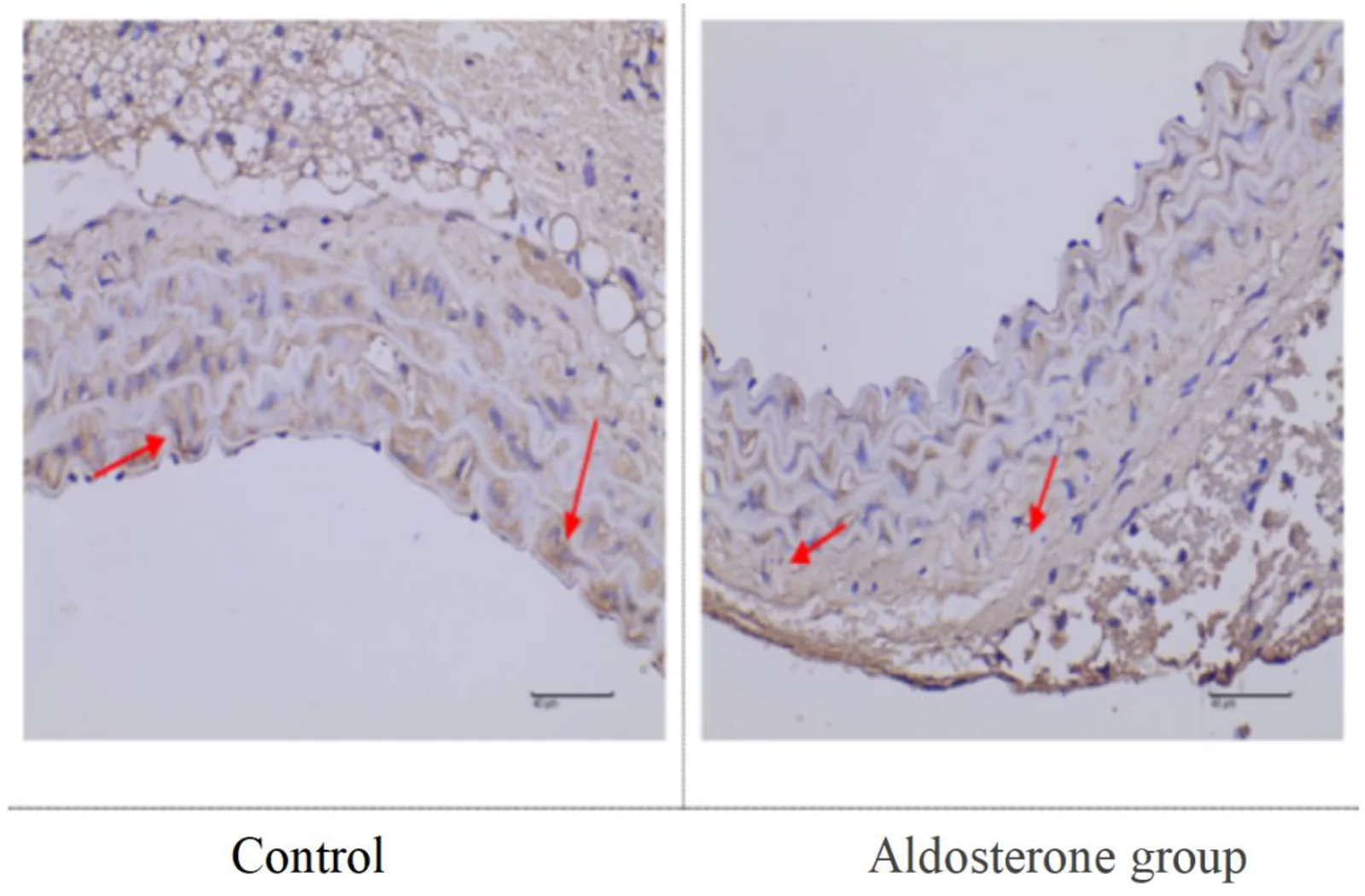
Representative immunohistochemistry (IHC) staining of TNX in mouse aortic tissue (n = 4 male C57BL/6J mice per group). Red arrows mark TNX-positive areas; scale bars are labeled on each slice; IHC, immunohistochemistry.

**Figure 10:**
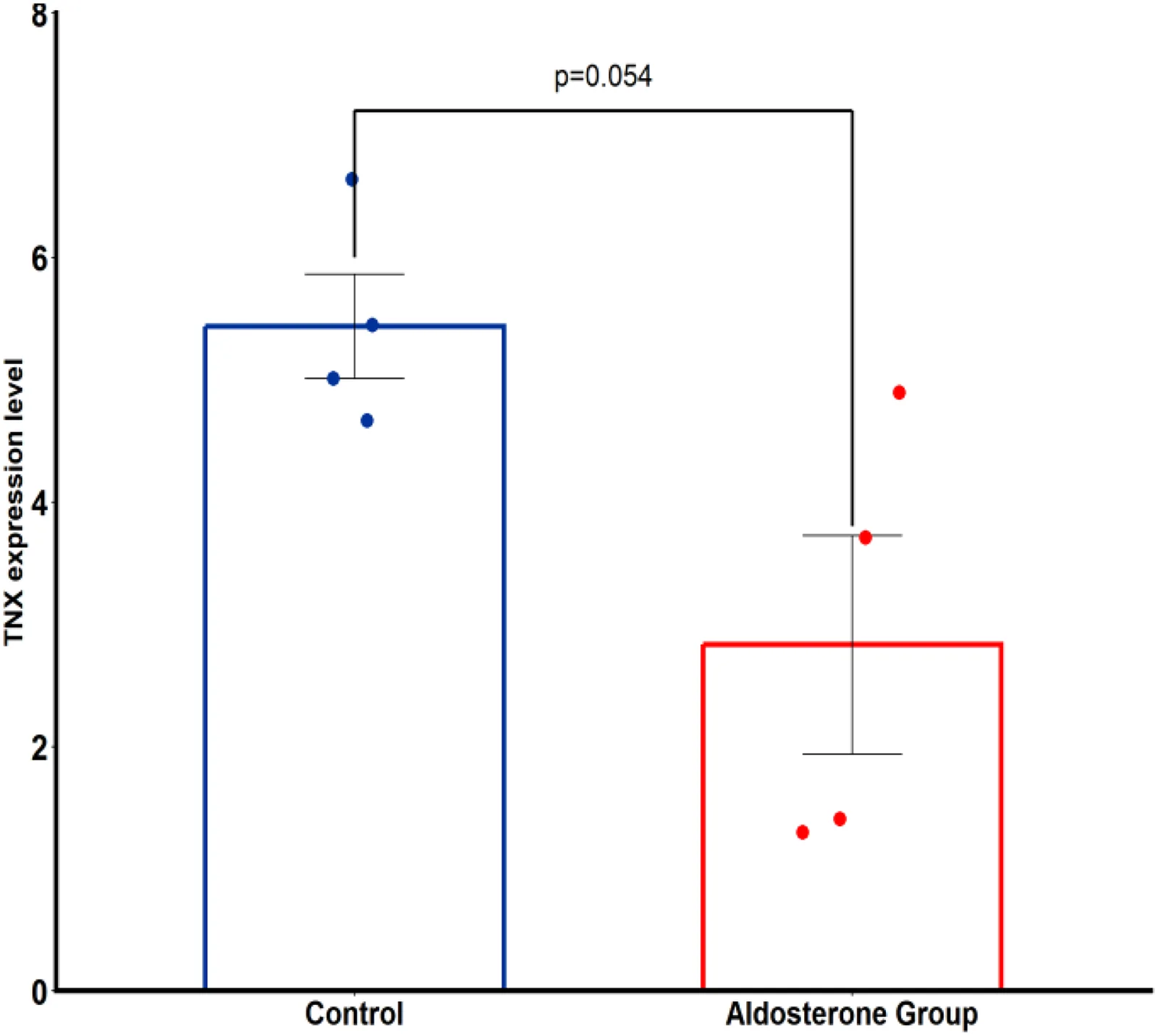
Quantification of TNX immunohistochemical positive area in mouse aortic tissue (n = 4 male C57BL/6J mice per group). No statistically significant difference was observed (*P* = 0.054), showing a borderline non-significant decreasing trend of TNX in aldosterone intervention mice. Bar plots display mean ± SD, and hollow scatter dots overlaid on bars represent the expression value of each individual biological replicate.

## 4 Discussion

This study employed DIA quantitative proteomics mass spectrometry to identify proteomic variations in the blood of two patient cohorts: those with PA and AAC and those with PA without AAC. A total of 741 potential proteins were identified, with 38 exhibiting differential expression: 6 were up-regulated and 32 were down-regulated. Bioinformatics analysis revealed that the differentially expressed proteins were predominantly associated with calcium-binding, collagen binding, and various other molecular functions. The signaling pathways implicated primarily pertained to ECM receptor interactions, mucins, and adhesion plaques in cancer. The 25 proteins exhibiting the highest connectivity were selected utilizing the STRING database. All of these proteins exhibited down-regulation. Notably, the observed downregulation of TNX in PA patients with AAC was identified for the first time and has not been previously reported. Therefore, this protein was selected for further validation at both the cellular and animal levels. *In vitro* experiments further confirmed that TNX protein expression was significantly downregulated in the aldosterone-treated group (*P* < 0.001). A non-statistically significant declining trend of TNX was observed in mouse aortic tissue samples. In this study, DIA proteomic screening identified TNX as a differentially expressed serum protein in PA patients with AAC, and downregulation of TNX by excess aldosterone was significantly confirmed in cellular models, while animal experiments showed a non-significant decreasing trend. Since in-depth mechanistic investigations have not been performed in the present work, the exact role of TNX in the pathological cascade of calcification remains to be further clarified. TNX may play an important role in vascular calcification occurring in patients with PA; nevertheless, this remains to be confirmed in further investigations.

Vascular calcification refers to the deposition of hydroxyapatite within the cardiovascular system, leading to vessel stiffness and disruption of the vessel wall structure (28). Research has demonstrated that ectopic vascular calcification is closely linked to the dysregulation of phosphate homeostasis (29), which is influenced by various factors, including osteoblasts, osteoclasts, fibroblasts, and smooth muscle cells (29). It is strongly associated with an increased risk of cardiovascular diseases and all-cause mortality (30). Previous studies have suggested that vascular calcification (VC) is a passive degenerative process associated with the ageing of blood vessels (31). However, most current research indicates that vascular calcification is not solely a degenerative change driven by mechanical factors (31); instead, it arises from a complex cascade of cytokine responses to endothelial cell injury (31). Recent studies increasingly support the idea that VC is an active process regulated through various molecular signaling pathways (32). These pathways, in addition to traditional osteogenic signals, involve processes similar to bone metabolism and cartilage formation (7, 11), as well as the apoptosis of VSMCs and the release of matrix vesicles (33). Pathological studies have shown that vascular calcification in patients with PA is primarily characterized by medial calcification, which is associated with osteogenic transformation of VSMCs, increased alkaline phosphatase (ALP) activity, and dysregulated extracellular matrix metabolism (34). Therefore, further investigation into the mechanisms underlying vascular calcification is crucial for advancing the prevention and treatment of cardiovascular diseases.

Evidence from previous studies suggests that vascular calcification in patients with PA mainly consists of intimal calcification and intima-media calcification (8, 35, 36), the latter of which is mostly associated with the formation of hydroxyapatite crystals and remodeling of the extracellular matrix within the vascular wall of large and medium-sized arteries.

The adhesion patch and ECM signaling pathways were identified as being involved in the pathogenesis of abdominal aortic calcification in PA through KEGG pathway analysis. The aorta’s structural integrity is contingent upon the ECM and VSMC (37).

The ECM is a non-cellular three-dimensional network of various macromolecules, including collagen, with components bound to each other and connected to cell adhesion receptors, whose surface receptors transmit signals to cells to regulate multiple functions and maintain homeostasis *in vivo*, and the ECM is a dynamic structure that is constantly remodelled under normal and pathological conditions mediated by matrix-degrading enzymes (38). The majority of cells produce a sophisticated extracellular matrix crucial for sustaining proper cellular activity. The extracellular matrix is crucial for the development of calcified structures (39–41). In the physiological process of osteogenesis, certain extracellular matrix components are synthesized, establishing a distinct environment that facilitates the precipitation of calcium and phosphate ions as hydroxyapatite minerals (39–42). Vascular calcification often occurs in atherosclerosis, and calcification is a prevalent characteristic of both bone and several atherosclerotic lesions, with calcification creation exhibiting many histological similarities to bone growth (43). Research indicates that a matrix with suitable composition and structure may undergo calcification even in the absence of cells (42). Thus, physiological calcification appears to be based on the ability of specialized cells to secrete a complex extracellular matrix that facilitates mineralization. Previously, it was found (44) that calcified vascular cells (CVC), cloned from a subpopulation of bovine aortic smooth muscle cells, exhibited several osteoblastic features *in vitro*-aggregation into nodules, spontaneous formation of hydroxyapatite minerals, and production of osteogenic differentiation factors (45). The CVCs generated substantial quantities of extracellular matrix (45). Vascular calcification may signify the transformation of cells that typically display a smooth muscle phenotype into cells resembling osteoblasts (43).

Ashwini S. Pai et al. also showed that matrix remodeling is important in the initiation and progression of vascular as well as aortic valve calcification (46). Recent studies indicate that the stiffness of the extracellular matrix influences the calcification of VSMCs (47). Elevated matrix stiffness was seen to reduce actin monomer concentrations in the nucleus of vascular smooth muscle cells, leading to the de-repression of YAP and the activation of TEAD and RUNX2, hence enhancing osteogenic gene expression and calcification (47). Recent studies have revealed that alterations in the ECM induced by aging and metabolic stress can directly promote the differentiation of VSMCs into osteoblast-like cells, thereby accelerating vascular calcification (48). Under conditions of mineral stress, ECM calcification is mediated by extracellular vesicles (EVs), which facilitate the uptake of hydroxyapatite by VSMCs (48). This process triggers oxidative stress, DNA damage, and the expression of osteogenic genes (48). Additionally, senescent ECM further activates osteogenic pathways in VSMCs, while ECM glycosylation enhances calcification by increasing alkaline phosphatase activity through receptor for advanced glycation end-products (RAGE) signaling (48). This study underscores the pivotal role of the ECM microenvironment in vascular calcification and offers new insights for the development of targeted therapies for related vascular diseases. However, the role of the differentially expressed protein TNX in patients with PA complicated by AAC remains insufficiently investigated. Our study addresses this critical knowledge gap.

Regarding how the extracellular matrix leads to alterations in the phenotype of vascular smooth muscle cells that ultimately lead to vascular calcification, previous studies have suggested that three aspects may be involved (43): 1. The direct effect of matrix molecules on cells. 2. Matrix-binding growth factors may have an impact on calcification in addition to the direct effect that matrix molecules have on cells. 3. Integrins are a large category of heterodimeric transmembrane receptors that assist cells in attaching to the extracellular matrix.

TNX is a member of the tenascin family of ECM proteins (49) and plays a significant role in collagen deposition (50), collagen fiber formation (51), and the assembly of elastic fibers (52). Evidence suggests that TNX is involved in vascular injury, with TNX deficiency leading to Ehlers-Danlos syndrome, a connective tissue disorder characterized by skin hyperelasticity and vascular fragility (53). Skin biopsies from affected patients reveal severe abnormalities in elastic fibers and collagen, which are critical components of blood vessel structure (52). Consistent with this, downregulation of TNX has been observed in calcified aortic valves (54), aligning with our findings. Recent studies have elucidated the role of TNX in hemodynamically regulated atherosclerosis (55). Previous work reports that pathological disturbed shear stress suppresses endothelial TNX and accelerates atherosclerosis, while physiological laminar flow upregulates TNX via the KLF4 pathway to protect vessels (55). Endothelial-specific deletion of TNX in mice resulted in increased expression of inflammatory markers, and exacerbated atherosclerosis (55). These findings provide novel mechanistic insights into the prevention of vascular inflammation and atherosclerosis (55). However, the association between TNX and AAC in patients with PA remains unexplored. Our study addresses this gap and provides complementary evidence. These findings suggest that reduced TNX expression in the vascular wall during calcification triggers alterations in extracellular matrix components, which may compromise the structural integrity and normal function of the collagen matrix. Published studies focusing on calcified aortic valves have verified that downregulated TNX serves as a pathogenic factor (54), consistent with our results. Meanwhile, hemodynamic studies also verify that TNX loss under abnormal blood flow aggravates vascular lesions, while elevated TNX serves as a protective factor. TNX may play a crucial role in the progression of vascular lesions.

To avoid false-positive results from proteomic analysis, *in vitro* validation experiments were performed in this study to further confirm the downregulation of TNX expression. Furthermore, we selected aortic tissues from aldosterone-modeled mice as well as control mice (4 mice in each group) for the detection of the relevant proteins, and found that TNX expression in the aldosterone-intervened mice group showed a decreasing trend without statistical significance (*P* = 0.054). This concordant trend requires further confirmation in a larger-sample animal group.

A comprehensive review of the relevant literature, together with our clinical, cellular, and animal experimental findings, indicates that vascular calcification is not simply a degenerative process resulting from mechanical stimuli, but rather an actively regulated biological event driven by diverse molecular signaling cascades. Among the various contributing factors, aldosterone-mediated remodeling of the extracellular matrix, followed by phenotypic switching of vascular smooth muscle cells and subsequent calcification, represents a particularly noteworthy avenue for future inquiry. Nevertheless, the precise molecular mechanisms involved remain largely unknown and merit further exploration.

### 4.1 Strengths and limitations

#### 4.1.1 Strengths

This study has several notable strengths. To our knowledge, no previous study has reported a proteomic analysis of patients with primary aldosteronism complicated by abdominal aortic calcification. This study provides new insights into vascular calcification associated with primary aldosteronism. Using DIA-based proteomics combined with rigorous *in vitro* cellular assays and *in-vivo* animal intervention experiments, we identified TNX dysregulation in PA patients with AAC, and experimentally confirmed aldosterone-induced TNX suppression, offering a novel mechanistic direction for future investigations. First, this proteomic study targeting abdominal aortic calcification in the setting of primary aldosteronism, provides candidate molecular clues for future mechanistic research on PA-related abdominal aortic calcification. Second, the study employs DIA label-free quantitative proteomics, which offers wider proteome coverage and more stable quantification compared with conventional methods, allowing more comprehensive and accurate identification of differentially expressed proteins in serum. Third, the study design is well-standardized with strict control of confounding factors. The two patient groups were matched for age and duration of hypertension, minimizing bias and improving the validity and comparability of the results. Fourth, a stepwise validation system was established, moving from clinical proteomic screening to *in vitro* cellular verification and then to *in vivo* animal model confirmation. This multi-level cross-validation enhances the robustness and reliability of the findings. Fifth, bioinformatic analyses including functional and pathway enrichment were systematically performed to clarify the key role of extracellular matrix dysregulation in aldosterone-related vascular calcification. Serum TNX downregulation was observed in PA patients complicated by abdominal aortic calcification, while our cellular animal experiments further demonstrated aldosterone-driven TNX suppression. These findings lay a solid foundation for further mechanistic research.

#### 4.1.2 Limitations

There are several inherent limitations within the current research. First, this is a retrospective case-control study conducted at a single medical center, which only recruited patients diagnosed with primary aldosteronism without an independent cohort of essential hypertension patients as external controls. Under this design, it is hard to tell whether the decreased TNX level is a unique alteration triggered by excessive aldosterone, or a common vascular damage outcome caused by hypertension alone. To alleviate such confounding bias, we implemented pre-planned 1:1 matching for age and hypertension duration at participant enrollment, together with strict inclusion and exclusion criteria. Apart from serum triglyceride and HDL-C, the remaining baseline indicators showed no notable inter-group disparities. These measures partially offset the drawbacks of our research design and suggest that the observed protein variation is not attributed to blood pressure levels or the length of hypertensive history. Additional experimental groups will be arranged in follow-up trials to carry out more in-depth exploration. Furthermore, our serum proteomic analysis should be regarded as exploratory, and the reported P-values are nominal. Accordingly, these proteomic findings should not be regarded as definitive statistical evidence.

Second, only four mice were allocated to each group for animal validation, resulting in insufficient statistical power. This largely explains why TNX expression in mouse aortic tissues merely exhibited a marginal declining trend without statistical significance (P = 0.054). Nevertheless, a clear reduction tendency of TNX protein was detected in the aldosterone-treated group. This concordant trend requires further confirmation in a larger-sample animal group. Follow-up animal verification tests will enlarge the number of animals per group to strengthen statistical credibility and reconfirm the findings reported here.

Moreover, this work merely confirmed the differential expression pattern of TNX without elaborate molecular mechanistic exploration. It remains unclear whether TNX functions as an intermediate regulatory factor connecting aldosterone signals to vascular calcification, or just acts as an auxiliary indicator for clinical assessment. For this reason, ROC curve analysis was not performed to evaluate its diagnostic performance in the present study. Meanwhile, TNX can be detected in connective tissues throughout the body rather than being specifically expressed in the aorta. Further mechanistic research will be implemented in the future to clarify its pathological and clinical significance.

Of note, serum TG and HDL-C differed significantly between groups. Some apolipoprotein-related DEPs may be subject to lipid-driven confounding. However, TNX, our primary target, is an ECM-derived glycoprotein rather than apolipoprotein. Its suppression upon aldosterone exposure was validated in VSMC and mouse aortic tissues, mitigating lipid-related confounding concerns. Given limited sample size, proteome-wide covariate adjustment was not performed, and future validation in lipid-matched PA cohorts is warranted.

## 5 Conclusions

This exploratory proteomic study identifies an inverse correlation between circulating TNX and abdominal aortic calcification in patients with primary aldosteronism, laying a foundation and offering preliminary clues for subsequent mechanistic research on PA-related vascular calcification.

## Data Availability

The raw data supporting the conclusions of this article will be made available by the authors, without undue reservation.
