# Supplementary material for: Inverse association between the extracellular matrix protein Tenascin-X and concurrent abdominal aortic calcification in patients with primary aldosteronism
Source: Front Endocrinol (Lausanne). 2026 Oct 1;17:1900237. doi: 10.3389/fendo.2026.1900237 (PMC13668541; doi:10.3389/fendo.2026.1900237)
Supplement: Supplementary file 1 [file Table1.docx]

Table S1 Differentially expressed serum proteins identified by DIA proteomics

| Accession | Gene Name | FC | P-value |
| --- | --- | --- | --- |
| A0A075B6J2 | *IGLV2-33* | 2.41 | 0.00 |
| Q99439 | *CNN2* | 1.48 | 0.04 |
| Q6UWP8 | *SBSN* | 1.47 | 0.02 |
| P04430 | *IGKV1-16* | 1.46 | 0.03 |
| P01601 | *IGKV1D-16* | 1.39 | 0.02 |
| P02655 | *APOC2* | 1.26 | 0.04 |
| P05090 | *APOD* | 0.83 | 0.04 |
| P12111 | *COL6A3* | 0.83 | 0.01 |
| P51884 | *LUM* | 0.83 | 0.01 |
| P98160 | *HSPG2* | 0.83 | 0.01 |
| P43121 | *MCAM* | 0.82 | 0.00 |
| P22105 | *TNXB* | 0.81 | 0.04 |
| P55290 | *CDH13* | 0.81 | 0.01 |
| Q9NPH3 | *IL1RAP* | 0.81 | 0.02 |
| Q16853 | *AOC3* | 0.79 | 0.01 |
| Q9H6X2 | *ANTXR1* | 0.78 | 0.02 |
| P23142 | *FBLN1* | 0.77 | 0.00 |
| Q9UBX1 | *CTSF* | 0.77 | 0.02 |
| P27487 | *DPP4* | 0.76 | 0.01 |
| Q9Y5Y7 | *LYVE1* | 0.76 | 0.01 |
| Q9NQ79 | *CRTAC1* | 0.75 | 0.02 |
| P55285 | *CDH6* | 0.75 | 0.03 |
| O00391 | *QSOX1* | 0.74 | 0.03 |
| Q6UXB8 | *PI16* | 0.74 | 0.01 |
| P04746 | *AMY2A* | 0.74 | 0.00 |
| Q9NS71 | *GKN1* | 0.72 | 0.03 |
| Q7Z3B1 | *NEGR1* | 0.71 | 0.01 |
| Q12866 | *MERTK* | 0.69 | 0.02 |
| Q6UY14 | *ADAMTSL4* | 0.68 | 0.03 |
| Q14623 | *IHH* | 0.67 | 0.02 |
| P30740 | *SERPINB1* | 0.67 | 0.04 |
| Q9UBG0 | *MRC2* | 0.63 | 0.01 |
| P30043 | *BLVRB* | 0.62 | 0.02 |
| Q15848 | *ADIPOQ* | 0.60 | 0.01 |
| P11684 | *SCGB1A1* | 0.60 | 0.03 |
| A0A0C4DH30 | *IGHV3-16* | 0.60 | 0.02 |
| Q9UBQ6 | *EXTL2* | 0.53 | 0.00 |
| P04439 | *HLA-A* | 0.47 | 0.03 |

Notes: FC, fold change; DEP cutoff: FC ≥ 1.2 or ≤ 0.83, P < 0.05; Gene symbols are italicized; *TNX,* Tenascin-X, encoded by the *TNXB* gene.
